# Moving towards new targets in the pharmacological treatment of substance use disorders

**DOI:** 10.1192/j.eurpsy.2025.1003

**Published:** 2025-08-26

**Authors:** V. A. Voicu, O. Vasiliu, A. Ciobanu

**Affiliations:** 1 Carol Davila University of Medicine and Pharmacy; 2 Romanian Academy of Medical Sciences; 3 Romanian Academy; 4 Psychiatry Dept., Dr. Carol Davila University Emergency Central Military Hospital; 5 “Prof. Al. Obregia” Psychiatry Clinical Hospital, Bucharest, Romania

## Abstract

**Introduction:**

Substance use disorders (SUDs) are a highly prevalent and complex class of psychiatric illnesses that lead to increased rates of mortality and morbidity, societal costs, low quality of life, and difficulties in all important areas of personal functioning. Although SUDs are constantly explored from a therapeutic perspective, there are very few validated treatments available for these disorders.

**Objectives:**

To review the evidence supporting new targets for the psychopharmacological management of patients diagnosed with SUDs.

**Methods:**

A literature review was conducted in three electronic databases (PubMed, Cochrane, and Clarivate/Web of Science) and the US National Library of Medicine database for clinical trials (www.clinicaltrials.gov) to find clinical and preclinical sources published between January 2000 and September 2024. The keywords used were „substance use disorders”, „drug dependence”, „drug addiction”, „new treatments”, „drugs in pipeline”, and „pharmacological treatment”. There was no restriction regarding the age of participants, and only sources published in English were selected.

**Results:**

Based on 12 sources identified for detailed analysis, the main directions of research in the field of new treatments for SUDs are modulators of glutamatergic and GABA-ergic systems, neurokinin-1 receptors, alpha-2 adrenergic agonists, G-protein signaling-biased μ-opioid receptor agonists, serotonin modulators, cannabinoid agonists or partial agonists, N-acetylcysteine, and monoclonal antibodies. These agents are explored for cocaine, opioids, nicotine, psychostimulants, and cannabis use disorder. Examples of investigational products from these emergent classes are SR-170189 (a biased mu-opioid receptor agonist without evidence of tolerance in preclinical studies), dexmedetomidine (BXCL501, an alpha-2 preferring agonist explored for opioid withdrawal), guanfacine (another alpha-2 agonist explored in cannabis use disorder-CUD), lorcaserin (a selective 5HT2C receptor agonist also explored for CUD), oxytocin (explored for alcohol use disorder- AUD and as add-on to methadone for comorbid opioid and cocaine use disorder), N-acetylcysteine (investigated for AUD and nicotine use disorder), NT-814 (neurokinin-1 and -3 receptor antagonist, investigated for opioid use disorder), memantine (a noncompetitive N-methyl-D-aspartate antagonist explored in patients with AUD), baclofen (a GABA-B receptor agonist explored for AUD, opioid withdrawal, and psychostimulants use disorder) and various biological therapies for NUD and cocaine use disorder.

**Conclusions:**

There are hopes related to an extensive number of investigational products that undergo different stages of clinical research for a large variety of SUDs, targeting multiple neurotransmitters and hormonal pathways, and even biological therapies that could block the action of the drugs of abuse before reaching their targets.

**Disclosure of Interest:**

None Declared

